# Urolithin A and nicotinamide riboside differentially regulate innate immune defenses and metabolism in human microglial cells

**DOI:** 10.3389/fnagi.2024.1503336

**Published:** 2024-11-27

**Authors:** Helena Borland Madsen, Claudia Navarro, Emilie Gasparini, Jae-Hyeon Park, Zhiquan Li, Deborah L. Croteau, Vilhelm A. Bohr

**Affiliations:** ^1^Center for Healthy Aging, Department of Cellular and Molecular Medicine, University of Copenhagen, Copenhagen, Denmark; ^2^Section on DNA Repair, National Institute on Aging, Baltimore, MD, United States; ^3^Computational Biology and Genomics Core, Laboratory of Genetics and Genomics, National Institute on Aging, Baltimore, MD, United States

**Keywords:** nicotinamide riboside, urolithin A, microglia, mitochondrial health, innate immune signaling, aging

## Abstract

**Introduction:**

During aging, many cellular processes, such as autophagic clearance, DNA repair, mitochondrial health, metabolism, nicotinamide adenine dinucleotide (NAD+) levels, and immunological responses, become compromised. Urolithin A (UA) and Nicotinamide Riboside (NR) are two naturally occurring compounds known for their anti-inflammatory and mitochondrial protective properties, yet the effects of these natural substances on microglia cells have not been thoroughly investigated. As both UA and NR are considered safe dietary supplements, it is equally important to understand their function in normal cells and in disease states.

**Methods:**

This study investigates the effects of UA and NR on immune signaling, mitochondrial function, and microglial activity in a human microglial cell line (HMC3).

**Results:**

Both UA and NR were shown to reduce DNA damage-induced cellular senescence. However, they differentially regulated gene expression related to neuroinflammation, with UA enhancing cGAS-STING pathway activation and NR displaying broader anti-inflammatory effects. Furthermore, UA and NR differently influenced mitochondrial dynamics, with both compounds improving mitochondrial respiration but exhibiting distinct effects on production of reactive oxygen species and glycolytic function.

**Discussion:**

These findings underscore the potential of UA and NR as therapeutic agents in managing neuroinflammation and mitochondrial dysfunction in neurodegenerative diseases.

## 1 Introduction

Inflammation causes many untoward effects in the disease pathophysiology of neurodegenerative diseases including Alzheimer's disease (AD), Parkinson's disease, and others. It is of paramount importance that the scientific community identify and characterize agents that can modulate inflammatory processes. Gratefully, many naturally occurring compounds exist and are currently under evaluation.

Urolithin A (UA) is a naturally occurring compound produced by gut bacteria from ellagitannins and ellagic acid, which are intricate polyphenols present in foods like pomegranates, berries, and nuts (D'Amico et al., 2021). UA was discovered 40 years ago (Doyle and Griffiths, 1980) and is considered the most conserved and studied urolithin across species (Tomás-Barberán et al., 2017), but only recently has its impact on aging and diseases been explored. UA extends the lifespan of *Caenorhabditis elegans* (*C. elegans*) and safeguards against physiological decline, as illustrated by improved muscle function in young animals and the prevention of age-related muscle decline in old mice, partially mediated by enhanced mitophagy (Ryu et al., 2016). UA also enhances cellular health by increasing mitophagy and mitochondrial function (Ryu et al., 2016; Fang et al., 2019b; Luan et al., 2021; Hou et al., 2024). Moreover, it also attenuates senescence (Cho et al., 2022). We previously reported that short-term (2 months) UA supplementation induced mitophagy in AD mouse brain, AD nematodes, and improved learning and memory in AD mouse models (Fang et al., 2019b). Our results were later validated by another study (Gong et al., 2019). Mitophagy genes, including PTEN-induced kinase 1 (*pink-1*), pleiotropic drug resistance 1 (*pdr-1*), and NIP3 homolog (*dct-1*), play important roles in ameliorating memory impairments and prolong the lifespan of *C. elegans* (Palikaras et al., 2015; Fang et al., 2019b). In line with this, UA effectively increased levels of PINK-1, PDR-1, and DCT-1, highlighting the potential application of UA in AD therapy to improve mitochondrial function and health span (Luan et al., 2021). UA also reduces detrimental inflammation in nematodes (Ryu et al., 2016; Fang et al., 2019b; Luan et al., 2021). More recently, we have reported that UA targets the cathepsin Z enzyme, which is involved in lysosomal functions (Hou et al., 2024). UA shows anti-neuroinflammatory effects in activated microglia, supporting the potential neuroprotective role of UA in AD brains (Xu et al., 2018). Other studies have confirmed the anti-inflammatory properties of UA *in vivo* and *in vitro* (Gong et al., 2019; D'Amico et al., 2021). Owing to its promising properties in healthy longevity, there are an increased number of proposed clinical studies with UA. For example, human studies have shown that it is safe (Andreux et al., 2019), and that it improved muscle endurance (Liu et al., 2022; Singh et al., 2022).

Nicotinamide riboside (NR) is a precursor of nicotinamide adenine dinucleotide (NAD+), which is an important coenzyme in a multitude of cellular processes. NAD+ levels decline with age and are also lower in many pathological conditions or age-related diseases (Lautrup et al., 2024; Migaud et al., 2024).

Therefore, boosting NAD+ levels is considered a strategy for improving cellular functions and human health (Cercillieux et al., 2022). NAD+ is crucial for proper mitochondrial functions, in energy production, metabolism, and redox homeostasis (Yang and Sauve, 2016), as well as mitochondrial biogenesis (Lapatto et al., 2023) and mitophagy (Aman et al., 2020). Activation of sirtuins by NAD+ also enhances mitochondrial function and stress responses (Bosch-Presegué and Vaquero, 2014; Lin et al., 2018), improves DNA repair, and reduces inflammation (Elhassan et al., 2019). Meanwhile, maintaining high levels of NAD+ facilitates the functions of PARPs (poly ADP-ribose polymerases) enzymes that utilize NAD+ as substrates, and promote more efficient DNA repair facilitating genome stability (Fouquerel and Sobol, 2014; Ray Chaudhuri and Nussenzweig, 2017; Lee et al., 2022). Moreover, enhanced NAD+ levels improve metabolic processes, enhance insulin sensitivity, and promote healthier lipid profiles, contributing to better overall metabolic health (Connell et al., 2019). Our previous studies showed that NAD+ supplementation by NR preserves learning and memory, maintains neuronal health, lowers DNA damage and inflammation in 3xTgAD/Polβ^+/−^ (Hou et al., 2018) and APP/PS1 (Fang et al., 2019b; Hou et al., 2021) AD mouse models, Werner syndrome patients, and animal models (Fang et al., 2019a), as well as Ataxia telangiectasia (A-T) models (Fang et al., 2016; Yang et al., 2021).

**Figure 1:**
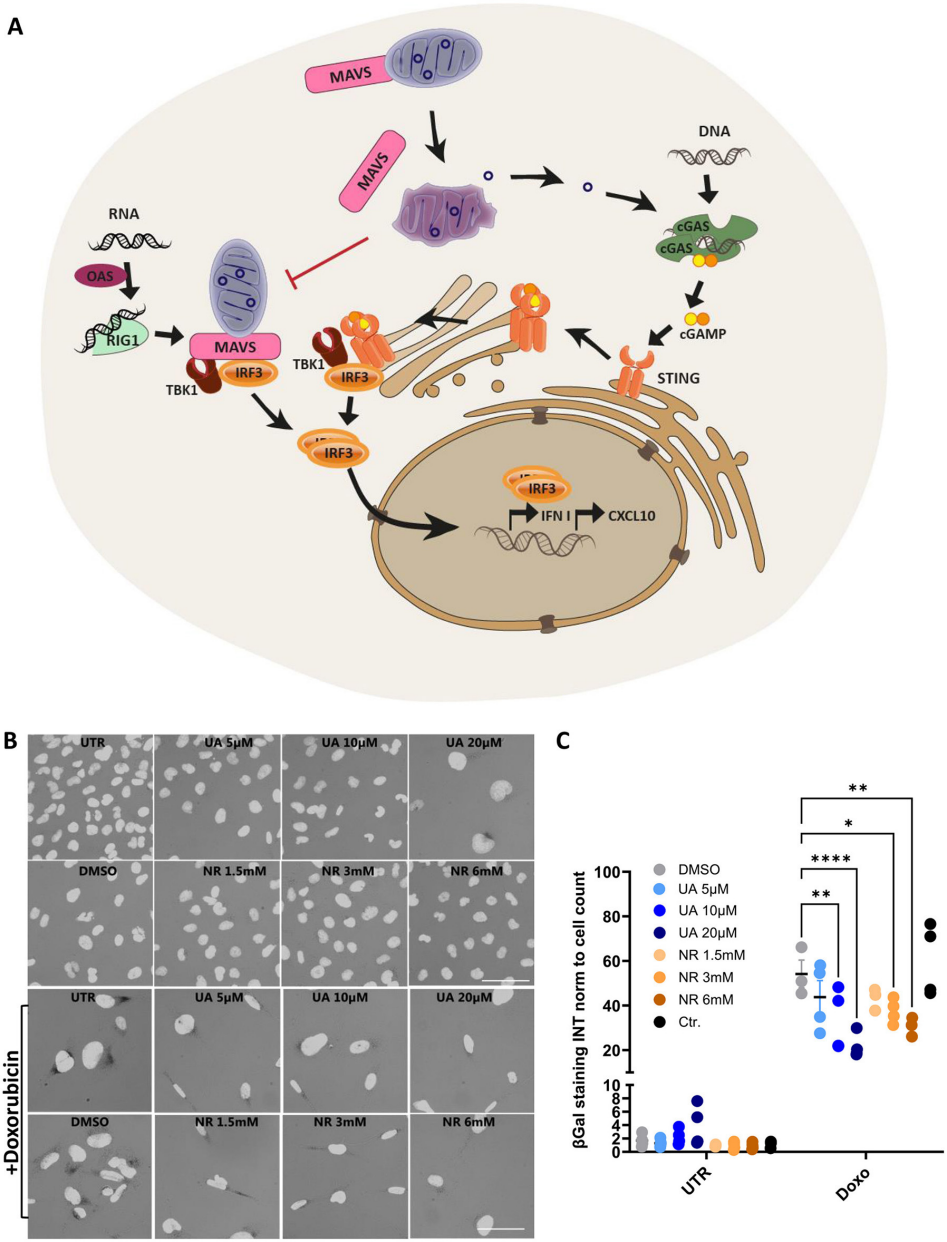
Schematic overview of the selected innate immune pathways and initial treatments with UA and NR decreasing DNA-damage-induced senescence. **(A)** Cytoplasmic DNA, from invading pathogens or from endogenous sources such as disrupted mitochondria, binds to and activates cyclic-GMP-AMP synthase (cGAS) to produce the second messenger, 2′3′-cyclic-GMP-AMP (cGAMP). cGAMP then activates the Stimulator of Interferon Genes (STING), which dimerizes and translocate to the trans-golgi network to recruit TBK and IRF3. Upon activation by phosphorylation, IRF3 translocates to the nucleus to activate the transcription of interferon genes. Cytoplasmic RNA can be sensed by 2′-5′-oligoadenylate synthetases (OASes), which activates RNAse L to cleave it into appropriate ligands for RNA pattern recognition receptors such as retinoic acid-inducible gene 1 (RIG-I). RIG-I then activates MAVS, which, like STING, engages TBK1 and IRF3, and IRF3 then translocates to the nucleus to induce the transcription of interferon genes in a STING-independent manner (Leisching et al., 2017). **(B, C)** Microglia cells were treated with/without doxorubicin 100 nM for 24 h, then treated for 1 week with either UA (5-20 uM) or NR (1.5-6 mM). Cells were stained for β-galactosidase **(B)** and a macro was used to quantify the signal intensity pr. cell **(C)**. *P ≤ 0.05, **P ≤ 0.01, ***P ≤ 0.001, ****P ≤ 0.0001.

UA and NR share a combination of anti-inflammatory and mitochondrial protective mechanisms, thus making them interesting targets to investigate. Specifically, these drugs modulate the innate immune pathways, where mitochondria play a significant role as a regulatory hub (Figure 1A). There are several immune sensors that scavenge the host environment for pathogenic insults, but of particular interest are the nucleic acid sensors cyclic GMP-AMP synthase (cGAS) and retinoic acid-inducible gene I (RIG-I), as they can be stimulated by mitochondrial DNA and RNA, respectively, beside viral and bacterial nucleic acids (Tigano et al., 2021; Kim et al., 2023). The RIG-I pathway is especially interesting, as one of the central mediators are localized to the mitochondrial membrane, namely the mitochondrial anti-viral signalling protein (MAVS) (Seth et al., 2005), and MAVS signalling is inhibited by both excessive mitochondrial fusion (Shi et al., 2014) and fission (Huang et al., 2021). RNA-ligands for RIG-I activation are primed by oligoadenylate synthases (OAS) (Choi et al., 2015). Upon RIG-I activation, it undergoes conformational changes to allow MAVS binding and oligomerization. MAVS then recruits the transcription factor IRF3 via the tank-binding kinase 1 (TBK1), which subsequently translocates to the nucleus to induce transcription of type 1 interferon genes. Similarly, cGAS activates the downstream stimulator of interferon response cGAMP interactor 1 (STING) via the second messenger 2′3′-cyclic GMP-AMP (cGAMP), allowing STING translocation and activation of IRF3 via TBK1 (Zhang et al., 2019).

The current study compared the effects of UA and NR on immune signalling, mitochondrial performance, and microglial function in a human microglial cell line, to provide further information to guide the use of these natural substances as common supplements, as well as disease-regulating compounds.

## 2 Materials and methods

### 2.1 Cell line and maintenance

The HMC3 cell line present in this study was obtained from ATCC (CRL-3304) and was cultured in Minimum Essential Medium Eagle (Merck) containing 10% fetal bovine serum and 1% GlutaMAX (Thermo Fisher Scientific). Treatment with UA and NR were conducted for 1 week at concentrations of 10uM and 3mM respectively unless otherwise specified, as we have previously shown effects of UA at this concentration and timepoint (Madsen et al., 2024).

### 2.2 Stimulation with DNA, cGAMP and poly(I:C)

The vector pAcGFP1-Hyg-N1 (Clontech) was digested with EcoRI and AseI. The digestion products were mixed and purified using NucleoSpin Plasmid Mini Kit (Macherey Nagel), giving rise to a mixture of several DNA fragments of 236, 1,143, 1,604, 2,209, 2,811, and 3,639 bp. These fragments, termed “DNA” for simplicity, were used at 25 ng pr. 8 well for 12 h to stimulate the cGAS-STING pathway by transfection, using Lipofectamine 3000 (Thermo Fisher Scientific).

For cGAMP-mediated STING stimulation, 30 μM 2′3′-cGAMP (InvivoGen) was added to full medium. For poly(I:C)-mediated RIG-I stimulation, 0.1 μg/mL (InvivoGen, tlrl-picw) was added to cells using lipofectamine 3000 (Thermo Fisher Scientific).

### 2.3 RNA isolation and nanostring gene expression analysis

RNeasy Mini Kit (Qiagen) was used to purify total RNA. Complementary DNA was prepared using Maxima Reverse Transcriptase and Oligo dT (12–18) (Thermo Fisher Scientific- Life tech).

NanoString analysis was carried out using total RNA isolated from the human HMC3 cells treated with vehicle, DNA, NR, or UA treatment using human neuroinflammation panel (XT-CSO-MNROI1–12), which contains transcripts of 770 neuroinflammatory genes covering the core pathways and processes that define neuroimmune interactions, and 13 internal reference genes for data normalization. Purified RNA was diluted in nuclease-free water to 20 ng/uL. It was hybridized in CodeSet mix carrying hybridization buffer, Reporter Code Set, and Capture Probe Set at 65°C for 16–24 h in a thermal cycler. Hybridized samples were loaded onto the nCounter Prep Station for immobilization in the sample cartridge (NanoString Technologies, MAN-C0035–07). The Prep Station can process up to 12 samples per run in ~2.5–3 h depending on which protocol was used. Next, the nCounter Digital Analyzer which was a multi-channel epifluorescence scanner collected data by taking images of the immobilized fluorescent reporters in the sample cartridge with a CCD camera through a microscope objective lens. The results were downloaded from the nCounter Digital Analyzer in RCC files format. NanoString readout was analysed via the NanoString nCounter nSolver 4.0 software (MAN-C0019-08) with the NanoString Advanced Analysis 2.0 plugin (MAN-10,030-03) according to the NanoString Gene Expression Data Analysis Guidelines (MAN-C0011-04). Both positive control and housekeeping normalization were used to normalize all sources of variation associated with the platform. Detection thresholds were established at the Log2 ratio relative to the reference (Log2 fold change). Genes with a fold-change cut-off of ≥|1.25| and *p*-value <0.05 were considered statistically significant. NanoString pathway score analysis was calculated as the expression values of constituent genes of each pathway via the NanoString nCounter nSolver 4.0 software. GEO access number is GSE266162.

### 2.4 Immunofluorescence

Cells were grown on Poly-L-lysine (P1399, Merck) coated eight-chambered object glass (177402, Thermo Scientific) over night. Cells were then treated with DNA/cGAMP/Poly(I:C) as described above, washed in PBS and fixed 10 min in 4% paraformaldehyde (sc-281692, Santa Cruz) at room temperature. Cells were blocked with 2% BSA (0332, VWR) dissolved in PBS, and permeabilized in 0.1% Triton X-100 (T8787, sigma) in PBS.

Primary antibodies: IRF3 (sc-33641, Santa Cruz) and pSTING (50907S, Cell Signalling) were diluted in PBS containing 1% BSA and incubated with cells at 4°C over night. Secondary Alexa488- and Alexa568-conjugated goat-anti-mouse (A11029, Invitrogen) and goat-anti-rabbit (A11011, Invitrogen) antibodies and DAPI (D9542, Merck) were incubated at room temperature with cells for 1$\frac{1}{2}$ h. Slides were then mounted (S3023, Dako) and imaged with an upright Leica DM4B microscope with a Leica EL6000 external light source and a Leica DFC365 camera. Images were analysed using an ImageJ (Version v1.53m) macro. The macro utilizes the DAPI channel to identify nuclear regions and measures the intensity of the IRF3 signals. The essential part of the code is presented in the supplementary material. Results are presented as the percentage of nuclei positive for IRF3 staining above a certain threshold, calculated using Microsoft Excel software. The pSTING signal intensity was measured and normalized to the number of cells per frame. GraphPad Prism V9.2 was used for plotting and performing two-way ANOVA analysis. Significance is scored as ns *p* > 0.05, ^*^p < 0.05, ^**^p < 0.01, ^***^p < 0.001. The number of biological replicates, n, corresponds to the number of datapoints in each figure.

### 2.5 Western blot

Cells were washed once in PBS before being scraped off and spun down. The cell pellet was lysed in RIPE buffer with phosphatase and proteinase inhibitors, and benzonase on ice for 30 minutes with regular mixing. Cells lysates were then spun down at 15,000 g for 30 min, and supernatant harvested for further analysis.

Protein extracts were separated in Tris-glycine SDS gels and transferred by blotting onto PVDF membranes. Membranes were blocked in 5% milk, before staining overnight with STING (13647 S), Rig-I (D12, sc-376845), p21 (Merck, SAB5700189), TREM2 (Cell Signalling Technology, #CST-91068S) and Actin (A5441) antibodies, using the prestained Ab116027 as molecular weight marker. ImageJ was used to quantify band intensities.

### 2.6 Reagents

Urolithin A (Merck), nicotinamide riboside (ChromaDex), Antimycin A (AA, cat. A8674), carbonyl cyanide 4-(trifluoromethoxy)phenylhydrazone (FCCP, cat. C2920), dimethyl sulfoxide (DMSO, cat. D8418), eagle's minimum essential medium (cat. M2279), L-glutamine (cat. G3126), monensin (cat. M5273), oligomycin (cat. 75351) and rotenone (cat. R8875), were obtained from Merck (St. Louis, USA). Seahorse XFe96 consumables were obtained from Seahorse Bioscience.

### 2.7 Mitochondrial oxygen consumption and glycolytic function

HMC3 human microglia cell line (ATCC CRL-3304) untreated or treated with 10 μM UA or 3 mM NR for a period of 1 week were plated at 2.2 x 10^4^ cells/well in a 96-well polystyrene Seahorse V7-PS Flux plate (XF plate). After a period of 24 h for cells adhesion, the wells were washed with XF base medium (cat 103334-100) supplemented with 11 mM glucose, 2 mM glutamine and 1 mM pyruvate (pH 7.4, assay medium). Then, the XF plate was pre-incubated in the assay medium for 1 h at 37°C in air. Measurements of oxygen consumption rates (OCR) were performed at 37°C using XF^e^96 Analyzer (extracellular flux, 96-well plate, Seahorse Bioscience). Previous reports have shown that oligomycin may underestimate maximal mitochondrial respiration (Ruas et al., 2016, 2018). Thus, to avoid misinterpretations of data, two independent experimental approaches were carried out. In the first experimental approach, basal OCR was measured, followed by the addition of oligomycin (1 μg/ml) to determine respiration-driven ATP synthesis and proton leak-linked OCR. In the second experimental condition, cells were allowed to reach the maximal respiratory capacity, after sequential additions of 200 nM of carbonyl cyanide 4-(trifluoromethoxy)phenylhydrazone (FCCP) but in the absence of a previous addition of oligomycin. The fraction of non-mitochondrial oxygen consumption was determined in both protocols after the addition of 1 μM rotenone plus 1 μM antimycin A.

Measurements of glycolytic rate and maximum glycolytic capacity in HCM3 cells under different treatments were performed based on the evaluation of the ECAR using XF^e^96 Analyzer (extracellular flux, 96-well plate, Seahorse Bioscience). The experiments were conducted according to a previously published protocol (Mookerjee et al., 2016). Thus, basal and maximal measurements of ECAR were assessed after sequential injections of 10 mM glucose followed by 1 μM rotenone plus 1 μM antimycin A, and then 100 μM monensin plus 1 μM FCCP.

The values were calculated in the Seahorse WAVE software from the absolute oxygen consumption rate (pmol O_2_/min) and extracellular acidification rate (mpH/min) measured in each evaluated parameter. Values were normalized by staining cells with 0.05% violet crystal dye (cat. CI42555, Merck) to determine cell number. The data shown in Figure 4 were acquired from the rate measurement equation, according to the manufacturer's instructions. To obtain the data presented in Figure 4, the separation of total extracellular acidification between respiratory proton production rate (PPR_resp_), and glycolytic proton production rate (PPR_glyc_) was carried out using the equation previously described (Mookerjee et al., 2015). The calculations were performed considering glutamine and glucose as cellular substrates.

### 2.8 Fluorescence-activated cell sorting (FACS) of mitochondrial ROS

HMC3 cells were seeded in 12-well plates (3^*^10^4^ cells/well) and incubated with DMSO control, 10μM UA, or 3mM NR for 6 days. After treatment, some DMSO control cells were treated with 20μM FCCP for 10min as a positive control. Cells were harvested for MitoSOX (Invitrogen, #M36008) (1 uM, 15 min) staining, followed by FACS analysis by CytoFLEX. FACS data were analysed by FlowJo and Prism 10.

### 2.9 β-galactosidase assay

The microglia cells, HMC3, were treated with different concentrations of UA (5–20 uM) or NR (1.5–6 mM) with/without 100 nM doxorubicin. After 6 days cells were stained for β-galactosidase activity according to the well-established protocol (Dimri et al., 1995). In short, cells were washed with PBS, fixed in 2% formaldehyde + 0.2% glutaraldehyde for 3 min, then washed again. Staining solution was incubated with cells for ~20 h. Cells were then stained for 5 min. with DAPI nuclear staining. An ImageJ macro was setup to quantify the number of cells and the intensity of the β-galactosidase staining, the essential part of the code is provided in the Supplementary material.

### 2.10 TAMRA-DNA degradation assay

We prepared TAMRA-tagged PCR-based DNA substrates for microscopy analysis. The substrates contained TAMRA at both ends (PCR3). Each PCR mix (50 μl) contained; 10 pmol of each primer, 200 μM of each dNTP, 2.5 mM MgCl2, 1 U Dream Taq PCR polymerase (Thermo Fisher Scientific), and 100 ng DNA template (pGEM-3Zf+, Promega). Amplicon 1.967 kb. PCR profile; 94°C 1 min, then 35 cycles of 94°C 30 s, 55°C 30 s, 72°C 1.5 min, and a final step72°C for 3 min. The PCR products were purified using QIAamp DNA Mini Kit (QIAGEN). HMC3 cells were treated with UA as described above. Before addition of TAMRA-tagged DNA, medium was changed to HBSS with calcium and magnesium, without Phenol Red (Cytiva) supplemented with 20 mM HEPES (Sigma) and imaged every 90 min using the Incucyte^®^ Zoom live cell analysis system. A basic analysis using the Incucyte^®^ Zoom software was conducted to score the total red object integrated intensity (RCU x μm^2^/image) over time.

### 2.11 Statistical analyses

The results are shown as representative and/or as the mean ± SEM. In a dot plot, each dot represents an independent experiment. ANOVA multiple comparisons test was used to assess differences between groups. Graph Pad Prism v10 was used to plot and conduct statistical analyses. Significance is scored as ns *p* > 0.05, ^*^p < 0.05, ^**^p < 0.01, ^***^p < 0.001.

## 3 Results

### 3.1 UA and NR decrease DNA damage-induced senescence

The human microglia cell line, HMC3, was used to model immune cells of the brain as it expresses key proteins of the immune response (Madsen et al., 2023). As both UA and NR have been shown to ameliorate several age-related processes, our initial studies explored their effectiveness in rescuing cells from damage-induced senescence, a state of cellular arrest, known to accumulate in aged tissues and aging-associated diseases (Di Micco et al., 2021).

HMC3cells were treated with different concentrations of UA (5–20 uM, blue) or NR (1.5–6 mM, orange). After 6 days, cells were stained for β-galactosidase activity, an agent commonly used to identify senescent cells (Figure 1B). There was no significant increase in the intensity of 5,5′-dibromo-4,4′-dichloro-indigo, the product quantified after cells were fixed and treated with X-gal, which is proportional to the β-galactosidase activity (Figure 1C, UTR). This was expected as UA and NR have been proven safe and should not induce senescence on their own (Andreux et al., 2019; Connell et al., 2019). However, there was a tendency that some β-galactosidase activity is activated at the highest concentration of UA, where the cells also seem somewhat enlarged (Figure 1B). The cytotoxic agent, doxorubicin was used to induce DNA damage, and thus senescence, which increased the β-galactosidase activity ~60-fold compared to untreated cells (Figure 1C, Doxo). Interestingly, when doxorubicin treatment was followed by UA or NR treatment, both UA and NR significantly and dose-dependently decreased β-galactosidase staining compared to the DMSO control. Thus, suggesting both have the capacity to decrease senescence development after stimuli.

In the following studies, we chose the middle concentration tested of both UA and NR, 10 μM UA and 3 mM NR, as these concentrations showed similar mean reduction in damage-induced senescence and generated no β-galactosidase activity on their own.

### 3.2 UA and NR differentially regulate gene expression in microglia cells

**Figure 2:**
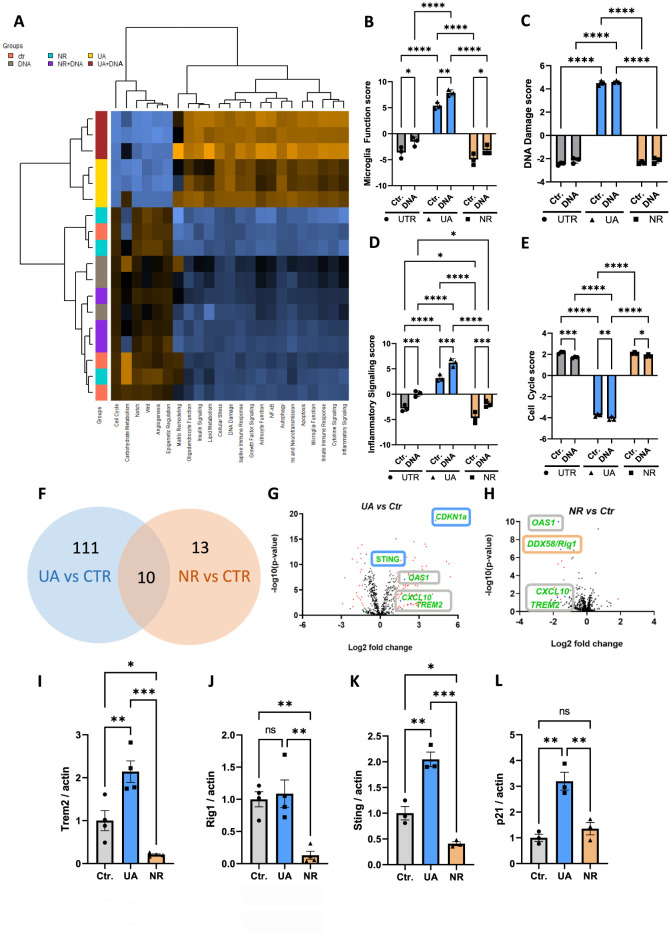
UA and NR differentially regulate transcription of several genes in microglia cells. **(A)** NanoString analysis was done to study neuroinflammation-related genes. Heatmap showing the directed global significance scores: orange denotes gene sets whose genes exhibit increased differential expression with the covariate, blue denotes gene sets with less differential expression. **(B–E)** Individual NanoString pathway scores following UA, NR, and/or DNA stimulation of HMC3 cells. **(F)** Venn diagram comparing the differentially expressed genes of UA vs. controls and NR vs. controls. There were ten genes that were in common, 111 UA subgroup-specific genes and 13 NR subgroup-specific genes. **(G, H)** Volcano plot representation of differential gene expression analysis of UA vs. controls and NR vs. controls. **(I–L)** Representative western blot analysis showing changed level of the TREM2, RIG-I, STING, and p21 following UA and/or NR treatment of HMC3 cells compared to control (UTR), analysed by two-way ANOVA. Each dot represents an independent experiment and bars denote means (+ SEM) (*N* = 4). **P ≤ 0.01 *vs*. untreated cells, Ctr.; *P ≤ 0.05 *vs*. untreated cells, Ctr. ***P ≤ 0.001, ****P ≤ 0.0001.

To further our analysis on the effects of UA and NR, RNA was harvested and subjected to gene expression analysis using NanoString technologies (see Methods for details). To investigate pathways and gene expression changes significantly affected by UA and NR in the microglia cell line HMC3, we employed the NanoString human neuroinflammation panel. This panel contains 770 genes associated with neuroinflammation and 23 neuroinflammatory pathways. The heatmap shows gene set analysis (GSA) with directed global significance scores and the expression profiles that were significantly different between UA and NR treatment (Figure 2A). GSA scores are essentially an average of the significance measures across all the genes in the pathway, as calculated by the differential expression. Orange colour indicates that the expression level was increased. Blue colour indicates that the expression level was decreased. Multiple inflammatory-related signalling pathways scores were modulated after treatment with NR and UA (Figures 2B–E, Supplementary Figure S1). Interestingly, UA and NR did not show the same response to the DNA stimulus. Pathways involved in microglia function, DNA damage, inflammatory signalling (Figures 2B–D), innate immune response, adaptive immune response, NF-kB signal, cytokine signalling, and apoptosis were significantly upregulated (Supplementary Figure S1), while cell cycle genes (Figure 2E) were significantly downregulated in the UA treatment compared to NR-treated groups.

We next identified 10 commonly regulated genes that were shared among both UA and NR subgroups. According to the NanoString neuroinflammation pathway panel, the following genes were significantly changed: *CCL5, CTSS, CXCL10, IFIH1, IL15RA, OAS1, PYCARD, RSAD2-, TRAF1, and TREM2* (Figure 2F). Interestingly, expression of several genes was differentially regulated by both UA and NR compared to control cells (Figure 2F), but in different directions, also visualized in the volcano plots (Figures 2G, H). Among these common genes, the Triggering receptor expressed on myeloid cells 2 (TREM2), OAS1 and C-X-C motif chemokine ligand 10 (CXCL10) were all upregulated by UA (Figure 2G) and downregulated by NR (Figure 2H). In contrast, STING and Cyclin Dependent Kinase Inhibitor 1A, known as p21, were upregulated only by UA (Figure 2G, blue boxes), and RIG-I was uniquely downregulated by NR (Figure 2G, orange box).

The gene expression analysis changes were verified with western blots of whole cell protein extracts. In agreement with gene expression changes, TREM2 protein levels were significantly increased by UA and decreased by NR (Figure 2I). Likewise, RIG-I protein levels were not altered by UA, but significantly decreased with NR (Figure 2J). STING protein levels were upregulated by UA and downregulated by NR (Figure 2K), consistent with the gene expression analysis. Finally, p21 protein was increased by UA, but not significantly affected by NR treatment (Figure 2L), again correlating with the gene expression analysis.

### 3.3 Innate immune pathways are differentially regulated by UA and NR

**Figure 3:**
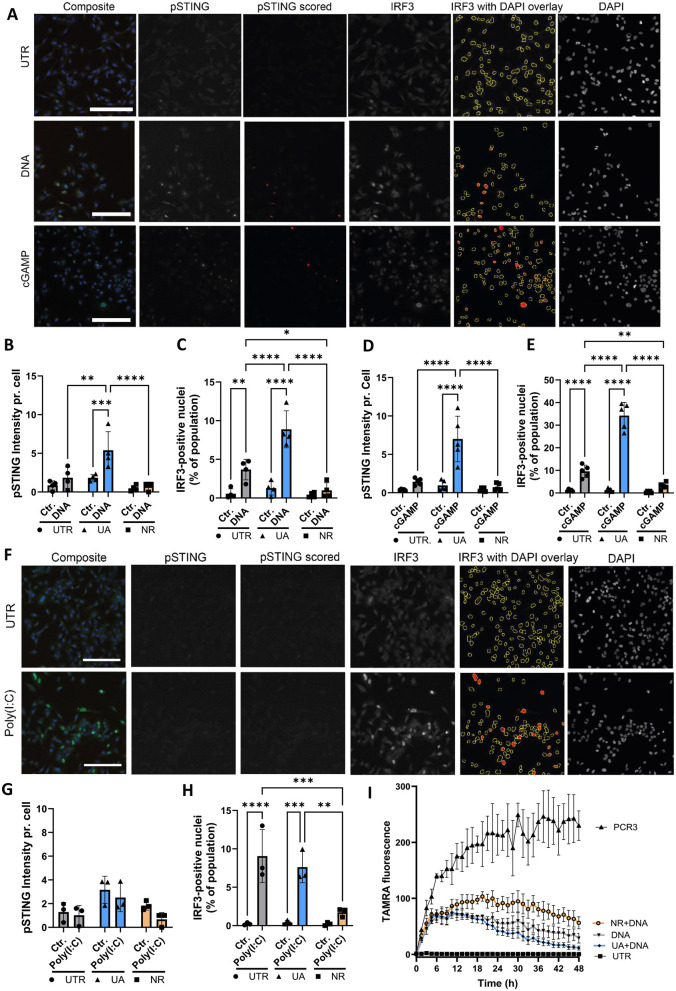
UA treatment leads to enhanced DNA stimulation, whereas NR treatment reduced both DNA and RNA signaling. **(A)** Immunocytochemistry imaging of control microglia cells treated with DNA or cGAMP and stained for DNA (DAPI), activated STING (pSTING) and IRF3. Scalebar = 250um. ImageJ macro analysis was used to quantify the amount of pSTING pr. cell **(B, D)** and the percentage of IRF3-positive nuclei **(C, E)**. **(F)** Images of microglia cells treated with the RNA mimic Poly(I:C) and stained as described above. pSTING and IRF3 translocation after Poly(I:C) were quantified in **(G, H)**, respectively. **(I)** Live-cell imaging of microglia cells degrading TAMRA-tagged DNA over 48 h. PCR3 is TAMRA-tagged DNA added to an empty well as a control. *P ≤ 0.05, **P ≤ 0.01, ***P ≤ 0.001, ****P ≤ 0.0001.

Since inflammatory signalling was differentially regulated by UA and NR (Figure 2), we sought to investigate this phenomenon further. In these experiments, microglia cells were stimulated with dsDNA using transfection, to achieve entry into the cytoplasm, as DNA can stimulate several different pathways, depending on entry and which receptors are engaged (endosome vs. cytoplasmic) (Kong et al., 2023). We focused on addressing activation of the cGAS-STING pathway because STING was upregulated by UA and downregulated by NR (Figure 2K). Immunofluorescence and semi-automated image analysis was applied to assess two central cGAS-STING activation checkpoints: phosphorylation of STING (pSTING) and nuclear IRF3 translocation (Figure 3A) upon stimulating cells with DNA to activate this pathway. Images are shown for the control cells with stimulations, and images of UA- and NR-treated cells are available in Supplementary Figure S2. From the images, the amount of activated STING (pSTING) increases, and the downstream transcription factor, IRF3, translocates to the nucleus, resulting in several bright nuclei, upon stimulation with dsDNA. The amount of pSTING per cell was significantly increased by pre-treatment with UA, before DNA stimulation (Figure 3B) in accordance with previous findings (Madsen et al., 2024). In contrast, pretreatment with NR had no effect on pSTING upon DNA stimulation, similar to the untreated (UTR) cells. Upon DNA stimulation, the percentage of IRF3-positive nuclei increased in UTR cells and was even more significantly increased upon treatment with UA (Figure 3C). Interestingly, NR treatment abolished IRF3 translocation upon DNA stimulation. Thus, two central activation signals in the cGAS-STING pathway, the early STING phosphorylation and the later IRF translocation, which is necessary to induce transcription of interferon genes, are promoted by UA pre-treatment and DNA stimulation, while NR downregulated the DNA-effect on IRF3.

Cells contain several DNA sensors besides cGAS (Kong et al., 2023), so in order to specifically investigate the cGAS-STING response, cells were also treated with the STING agonist, cGAMP, without transfection. Like the DNA-treatment, the cGAMP response was increased by UA pre-treatment both when looking at STING phosphorylation and IRF translocation, whereas NR pre-treatment had no effect on STING phosphorylation (Figure 3D) but significantly inhibited IRF3 nuclear translocation (Figure 3E).

Our NanoString analysis showed a general increase in expression of inflammatory signalling genes (Figure 2D), but curiously, we found that RIG-I, another intracellular receptor important for induction of type 1 interferon genes, was unaffected by UA and downregulated by NR on both the RNA (Figures 2G, H) and protein level (Figure 2J). To investigate this further, microglia cells were transfected with poly(I:C), an RNA mimic to stimulate the RIG-I/MAVS pathway (Figure 3F, Supplementary Figure S3 for a complete set of images). As pSTING is not directly activated in this pathway, pSTING staining was included as a control, and showed no staining upon poly(I:C) stimulation, as expected (Figure 3G). In accordance with RNA and western blot data, UA pretreatment did not affect the degree of IRF3-translocation compared to control cells, however, NR pretreatment significantly hampered the activation (Figure 3H, Supplementary Figure S3).

Collectively, these data support a general anti-inflammatory effect of NR pretreatment, while pretreatment with UA enhances the cGAS-STING pathway specifically, consistent with our prior publication (Madsen et al., 2024).

### 3.4 Degradation of DNA is differentially affected by UA and NR

Besides inducing inflammation, microglia are specialized scavengers, and have a high phagocytic capacity. Another interesting gene that is differentially regulated by UA and NR, is TREM2, which is upregulated by UA and downregulated by NR (Figures 2G–I). While TREM2 is an important innate immune receptor, and thus part of the inflammatory signalling, it has also been shown to be critical for microglia function, especially their phagocytic capabilities (reviewed in Li et al., 2023). Therefore, we tagged dsDNA with a TAMRA fluorophore, and followed the ability of our microglial cells to degrade these products (“PCR3”) over time using live-cell microscopy (Figure 3I). The black line labelled PCR3 shows TAMRA fluorescence in wells without microglia, to confirm that these PCR products were not non-specifically degraded by components in the culture medium. The black line labelled DNA shows increasing fluorescence during the first couple of hours as TAMRA-tagged fluorescent products sink to the bottom of microglia-covered wells, coming into focus, before the fluorescence start to decrease as cells have taken up and start to degrade these products. Interestingly, UA pretreatment results in a lower amount of substrate scored over time (blue line), whereas NR pretreated cell cultures scored more substrate at any time compared to UA and untreated cultures (orange line). These data suggest that UA seems to enhance cellular degradation of TAMRA-tagged DNA in line with a recent study (Hou et al., 2024), whereas NR treatment leads to slower removal of the substrate.

### 3.5 Mitochondrial dynamics are differentially affected by UA and NR

Finally, we sought to investigate the mitochondrial health in UA- and NR-treated cells. Besides driving the cells' energy production in the form of ATP, mitochondria are known to be master regulators of the inflammatory response, themselves containing damage-associated molecular patterns such as mitochondrial DNA, mitochondrial RNA, and ATP which can drive inflammation (Marchi et al., 2023). Furthermore, Rig-1 signals via the mitochondrial membrane protein MAVS and dissociation of the mitochondria disrupts Rig-1/MAVS signalling (Lin et al., 2006). In that sense, the health of a cell is directly proportional to the function and health of its mitochondria. ATP is the currency cells use to perform work and cellular respiration via oxidative phosphorylation and glycolysis are the primary mechanisms cells use to generate ATP. When mitochondria are superfluous or in bad shape, they are turned over by a process called mitophagy. Notably, both UA and NR have previously been shown to activate mitophagy and improve mitochondrial health (Fang et al., 2016, 2019b; Ryu et al., 2016; Aman et al., 2020; Luan et al., 2021; Lapatto et al., 2023). Mitochondria are also a major source of reactive oxygen species (ROS). Thus, we sought to investigate if UA and NR modulate oxygen consumption rates (OCR), glycolytic function, and mitochondrial ROS production.

**Figure 4:**
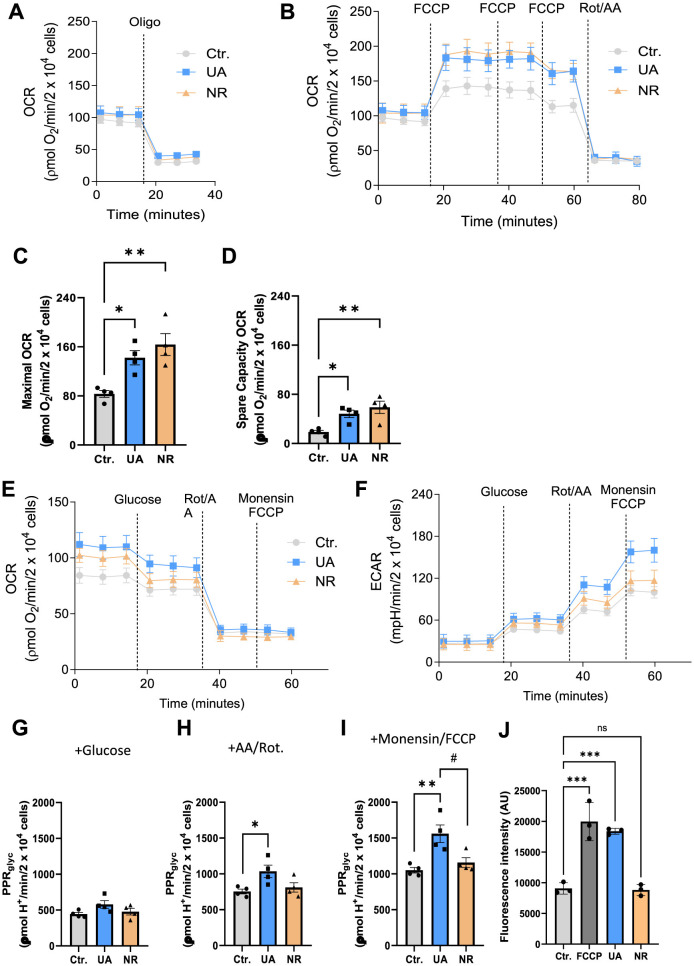
Treatment with UA and NR promotes a significant increase in maximal and spare capacity OCR in HCM3 cells, with maximal glycolytic function increased only by UA treatment. HCM3 cells (2.2 × 10^4^ cells/well) untreated (Ctr.) or treated with 10 μM UA or 3 mM NR for 1 week were incubated in appropriate medium containing 2 mM glutamine and 11 mM glucose as metabolic energy substrates for OCR measurement and 2 mM glutamine for PPR measurement. **(A, B)** show representative traces of oxygen consumption rates (OCR). Oligomycin (Oligo, 1 μg/mL), carbonyl cyanide 4-(trifluoromethoxy)phenylhydrazone (FCCP, 200 nM each addition), and rotenone (Rot, 1 μM) plus antimycin A (AA, 1 μM) were added where indicated. Different OCR parameters were determined: **(C)** Maximal respiratory capacity and **(D)** Spare respiratory capacity. For evaluation of glycolytic function, Panels E and F represent the experimental design and raw data of OCR and extracellular acidification rate (ECAR). Glucose (10 mM), rotenone (Rot, 1 μM) plus antimycin A (AA, 1 μM), and monensin (100 μM) plus FCCP (1 μM) were added where indicated. **(G)** Basal glycolytic proton production (PPRglyc) and maximal glycolytic rate stimulated by rotenone plus antimycin A **(H)** and monensin plus FCCP **(I)** were calculated from OCR and ECAR. Each dot represents an independent experiment, and bars denote means (+ SEM) (*N* = 4). **P ≤ 0.01 vs. untreated cells, Ctr.; *P ≤ 0.05 vs. untreated cells, Ctr. #*P* ≤ 0.05 vs. NR-treated cells. **(J)** HMC3 cells were treated with DMSO, 10 μM UA, or 3 mM NR for 6 days, respectively. The FCCP group was treated with 20 μM FCCP for 10 min and controlled for by using DMSO. Data was presented as mean with standard deprivation. Data were statistically tested using ordinary one-way ANOVA by Prism 10. ***P < 0.001.

OCR measurements were performed in cells previously treated with 10 μM UA or 3 mM NR for a period of 1 week. Figures 4A, B show representative traces of two independent approaches to measure OCR using mitochondrial inhibitors oligomycin (Oligo) and rotenone/antimycin A (Rot/AA) and the uncoupler Carbonylcyanide-p-trifluoromethoxyphenylhydrazone (FCCP). The protocol for Figure 4A was used to determine basal, ATP-linked, and H^+^ leak- linked OCR (Supplementary Figures S4A–C, respectively). Figure 4A, Supplementary Figure S4A shows that there was no difference in the basal respiration rate of HMC3 cells treated with the agents, NR and UA, compared to untreated cells (Ctr.). The inhibition of mitochondrial ATP synthase (Complex V) by oligomycin decreased basal respiration proportionally so there were no differences in ATP-linked respiration (Figure 4A, Supplementary Figure S4B). Likewise, treatment with NR and UA in HMC3 cells did not alter the H^+^ leak-linked OCR when compared to untreated cells (Supplementary Figure S4C).

In Figure 4B, maximal OCR and spare capacity OCR were determined by titration with the protonophore FCCP (Figures 4C, D, respectively). Interestingly, treatment with either NR and UA promoted a significant increase in maximal OCR (Figure 4C) in NR- (*p* = 0.0047) and UA-treated cells (*p* = 0.028) when compared to untreated cells. The spare respiratory capacity (Figure 4D, Supplementary Figure S4D), calculated by subtracting the FCCP-stimulated from the basal OCR, was also increased in cells treated with both NR (*p* = 0.008) and UA (*p* = 0.042) when compared to the untreated cells. Figure 4B, Supplementary Figure S4E show there were no differences in non-mitochondrial OCR between control or treated cells.

Oxidative phosphorylation and glycolysis are the main ATP-producing mechanisms in cells. Thus, we similarly measured glycolytic functions via Seahorse in the cells plus or minus UA and NR treatment. Cells were treated with 10 μM UA or 3 mM NR for a period of 1 week and then cellular glycolytic function was evaluated by simultaneous measurements of extracellular acidification rate (ECAR) and OCR. Figures 4E, F show the experimental design and raw OCR and ECAR data used for glycolytic proton production rate (PPR_glyc_) estimation. Figures 4G–I show the results of PPR_glyc_ under basal and maximal glycolytic rate stimulated by rotenone plus antimycin A and monensin plus FCCP, respectively. The basal glycolytic function was not altered due to the treatments with UA or NR Figure 4G. However, the data shown in Figure 4H demonstrate that only UA treatment increased maximal glycolytic function when compared to Ctr cells (*p* = 0.044). To avoid an underestimation of cellular glycolytic rate, we isolated glycolysis as the sole ATP producer and increased the cellular demand for ATP by using monensin, an ionophore that increases Na^+^/K^+^-ATP-ase and ATP demand (Mookerjee et al., 2016). The data in Figure 4I indicates that cellular ATP demand underestimates the maximal glycolytic rate in microglia cells by 30%, this effect being independent of the treatment carried out on the cells. Furthermore, a higher maximal glycolytic rate was found in UA-treated cells compared to the control group (*p* = 0.006) or the NR-treated cells (*p* = 0.023) (Figure 4I).

Mitochondria are also a major source of reactive oxygen species; (ROS), and usually, dysfunctional mitochondria produce more ROS. Therefore, mitochondrial ROS was determined by mitochondrial superoxide indicator (MitoSOX). FCCP treatment increased mitochondrial ROS generation. UA also elevated the ROS levels, whereas NR showed no effect on ROS in HMC3 cells compared to control cells (Figure 4J).

Although both UA and NR are recognized as important stimulators of mitophagy, these data emphasize that they affect mitochondrial physiology differently, as UA shows a significant impact on glycolytic function in microglial cells which NR does not recapitulate.

## 4 Discussion

The NanoString neuroinflammation panel analysis, Figure 2, revealed stark differences between UA- and NR-treated HMC3 cells. By this analysis, we find that UA-treated cells, both with and without DNA treatment, were distinctly different from control and NR treated cells (Figure 2A). NR- treated cells, with or without DNA treatment, were not well separated from the control cells, and clustered within the control group. Further the NR- and DNA-treated cells do not cluster together either. This suggests that only UA treatment strongly affected the pathways included in the NanoString neuroinflammatory panel. Generally, UA downregulated pathways associated with growth, and UA upregulated pathways associated with inflammation, autophagy and damage. This correlates with increased cGas-STING signalling upon DNA- and cGAMP treatment and increased clearance of TAMRA-tagged DNA.

UA has recently been shown to normalize AD-associated inflammation (Hou et al., 2024), and age-associated inflammation (Ignacio Jiménez-Loygorri et al., 2024) in mice, while significantly reducing C-reactive proteins in a human randomized trial via mitophagy stimulation (Singh et al., 2022). The anti-inflammatory effects of UA are well documented, thus our previous report on UA-induced STING expression in microglia cells and priming these cells for a stronger innate immune response via cGas-STING signalling (Madsen et al., 2024), was unexpected then, yet replicated in the current study. Interestingly, in the study by Jimenez-Loygorri et al. (Ignacio Jiménez-Loygorri et al., 2024), UA does seem to increase cGas-STING pathway genes in young mice compared to controls, whereas UA normalizes the expression in aged mice. This suggests a more complex role of UA, which may be both cell type- and age dependent. We have also shown that even though pro-inflammatory genes such as NF-kB and CXCL-10 are upregulated in the nanostring analysis, qPCR analysis showed no effect of UA on the CXCL-10 gene, and NF-kB activation was even reduced by UA in the same cells (Madsen et al., 2024), in line with previous reports (Komatsu et al., 2018; Abdelazeem et al., 2021). Importantly, UA does not induce inflammation on its own, even though UA tends to activate STING phosphorylation in certain studies (Madsen et al., 2024). This effect varied both in our previous study between assays, and in the current study, suggesting that the potential effect of UA alone on STING activation may be borderline. Nevertheless, the effect of UA on upregulating innate immune genes such as STING is robust. It is interesting to note that the primordial effect of STING is thought to be as a driver of autophagy (Gui et al., 2019), correlating nicely with the effect of UA to increase the clearance of TAMRA-tagged DNA. On the same note, NR downregulated STING protein levels, significantly decreased cGas-STING signalling and, in correlation, inhibited the cellular capability to degrade TAMRA-tagged DNA. This data suggests directly opposing effects of UA and NR in regulating the innate immune system, specifically when responding to cytoplasmic DNA. Further studies are necessary to identify if this effect is general to immune cells and whether it affects the cell's ability to combat e.g., DNA-viruses or remove bacterial DNA.

We further show that NR has opposing effects to UA, specifically downregulation of TREM2 and STING and inhibition of IRF-3 translocation upon DNA- and cGAMP stimulation. Further, NR shows a more general reduction in innate immune responses, as it also downregulated IRF-3 translocation upon stimulation with the RNA mimic, Poly(I:C), possibly via decreasing Rig-I RNA and protein levels. This is in correlation with another report showing that RIG-I is downregulated by NR- and NMN- (another NAD+ precursor) treatment in mice (Doke et al., 2023). Additionally, this study finds that NAD+ precursors ameliorate cisplatin-induced kidney dysfunction in mice by protecting mitochondria and thus prevent mitochondrial RNA leakage to the cytosol.

It was unexpected to find that UA decreased cell cycle genes, and increased DNA-damage associated genes and expression of p21, especially since the common β-galactosidase assay to visualize senescent cells, showed no significant increase for any substrate at the concentrations used here. Furthermore, both UA and NR dose-dependently decreased β-galactosidase staining after doxorubicin treatment. This is in line with earlier reports showing that NR (Yang et al., 2021) and UA (Cho et al., 2022) prevent cellular senescence. The process of cellular senescence is multifaceted and complex (Di Micco et al., 2021), and warrants further analyses, given these results.

Mitochondrial bioenergetics is becoming increasingly recognized as an important factor in regulation of the immune response. While basal glycolytic function was not affected by any of the treatments, UA increased the maximal glycolytic function and rate. Increased glycolysis has been associated with cytokine production and inflammation and is increased in microglia from the 3xTg mouse model of AD (Sangineto et al., 2023). However, the role of glycolysis in immune cell regulation and activation is cell type and context dependent (Pajak et al., 2024). An elevation in the maximal, but not basal function, seem to correlate with our previous study, showing that UA treatment does not initiate the innate immune response itself, but prepares microglia for a stronger response upon stimulation (Madsen et al., 2024).

The elevation of both maximal- and spare capacity OCR by UA and NR, reflect an improved capacity to produce energy in response to increased stress. Previous studies corroborate UA's role in promoting mitochondrial biogenesis in various models. For example, in *Caenorhabditis elegans*, UA treatment was directly associated with increased mitochondrial biogenesis and maximal OCR, leading to greater mobility and longevity (Ryu et al., 2016). Similarly, in human, UA treatment increased the expression of genes related to mitochondrial metabolism, accompanied by greater mitochondrial biogenesis in muscle samples from elderly individuals (Andreux et al., 2019; Singh et al., 2022). Our findings reinforce these results, suggesting that the improvement in maximal capacity following UA treatment appears consistent across experimental models and samples.

On the other hand, the effects of NR on mitochondrial bioenergetics have shown wide variability depending on the cell type, experimental model, and treatment duration. Liufu and colleagues (Liufu et al., 2023) reported that after 7 days of NR treatment in fibroblasts derived from patients with mitochondrial diseases, there was an increase in NAD^+^/NADH ratio and ATP levels; however, no significant changes were observed in maximal OCR, contrasting with the findings of the present study. This discrepancy may be related to intrinsic differences between fibroblasts and microglia, as well as the impact of oligomycin in underestimating maximal OCR. We showed that the presence of oligomycin significantly inhibited maximal OCR in microglia after NR treatment (Supplementary Figure S4E). These findings highlight the importance of considering the specific characteristics of each cell type and emphasize how experimental design can influence the data obtained when evaluating mitochondrial bioenergetics. In another study involving human neurons deficient in autophagy, and exhibiting cytotoxicity and mitochondrial dysfunction, NR treatment for 6 days restored intracellular NAD^+^ levels, increased basal respiration and maximal respiratory capacity (Sun et al., 2023). These results corroborate our findings on NR's beneficial effects on mitochondrial bioenergetics.

NAD+ (Khan et al., 2014; Schöndorf et al., 2018) and UA (Yang et al., 2023) supplementation have previously been reported to restore mitochondrial morphology in disease models. Interestingly, another study compared the NAD+ precursor nicotinamide mononucleotide (NMN) to UA in a prion disease model, and found that while both agents induced mitophagy, only the NAD+ precursor could alleviate prion-induced mitochondrial fragmentation (Li et al., 2022). Another study reported no structural mitochondrial changes in aged mice treated with NR, however there was no obvious defect reported comparing aged to young mice, leaving no window for a potential treatment effect in this study (Sun et al., 2021). These studies together indicate that the potential beneficial effects of these compounds on mitochondria are context dependent.

It is worth noting that, while UA and NR have improved mitochondrial function in a multitude of preclinical studies, the human studies generally fail to show clear improvements in mitochondrial function but support a reduction in some inflammatory markers (Damgaard and Treebak, 2023; Kuerec et al., 2024). This notion may be due to the relatively healthy individuals included in most of the human studies, vs. the severe phenotypes studied in the preclinical trials. In the current study, normal microglial cells are investigated which could explain the relatively mild to no effect observed by these treatments alone, while, after challenging the cells either with inducers of inflammation or the DNA damaging agent doxorubicin, both UA and NR show clear protecting effects. This discrepancy should be kept in mind for future studies and warrants further investigations.

Together with the existing literature, our findings suggest that both UA and NR exhibit therapeutic potential for improving mitochondrial bioenergetics across different cell types and experimental conditions. However, further research is needed to elucidate the underlying mechanisms.

While both UA and NR are regarded as safe and widely sold as anti-aging dietary supplements, their diverse roles on the cellular level are still being unravelled. Our data, along with previous studies, provide further information on the effects of UA and NR in this head-to-head comparison in microglia cells. Thus, the current study is important to guide the use of UA and NR both as common supplements, as well as disease-regulating substances.

## Data Availability

The datasets presented in this study can be found in online repositories. The names of the repository/repositories and accession number(s) can be found below: https://www.ncbi.nlm.nih.gov/genbank/, GEO access number is GSE266162.

## Supplementary material

Supplementary Figure S1 NanoString pathway scores from the analysis in Figure 2A following UA, NR, and/or DNA stimulation of HMC3 cells for the pathways Innate Immune Response **(A)**, Adaptive Immune Response **(B)**, Cytokine Signalling **(C)**, Apoptosis **(D)** and NF-kB **(E)**.

Supplementary Figure S2 Immunocytochemistry imaging of microglia cells treated with either UA or NR for 1 week, then stimulated with DNA or cGAMP and stained for DNA (DAPI), activated STING (pSTING) and IRF3. Scalebar = 250um. An ImageJ macro was used to quantify the pSTING (pSTING scored) and nuclear IRF signals (IRF3 with DAPI overlay) shown in red.

Supplementary Figure S3 Immunocytochemistry imaging of microglia cells treated with either UA or NR for 1 week, then stimulated with poly(I:C) and stained for DNA (DAPI), activated STING (pSTING) and IRF3. Scalebar = 250 um. An ImageJ macro was used to quantify the pSTING (pSTING scored) and nuclear IRF signals (IRF3 with DAPI overlay) shown in red.

Supplementary Figure S4 Mitochondrial bioenergetics parameters and the effect of oligomycin in the Spare Capacity OCR in microglia cells treated with either UA or NR for 1 week. HMC3 cells (2.2 x 10^4^ cells/well), untreated (Ctr.) or treated with 10 μM UA or 3 mM NR for 1 week, were incubated in appropriate medium containing 2 mM glutamine and 11 mM glucose as metabolic energy substrates for OCR measurement. Different OCR parameters were determined: **(A)** Basal OCR, **(B)** ATP-Linked dependent OCR, (C) H^+^ Leak-linked OCR, and (D) Non-mitochondrial OCR. Panel E shows the effects of oligomycin on the underestimation of Spare Capacity OCR in cells subjected to different treatments. Each dot represents an independent experiment, and bars denote means (+ SEM) (*N* = 4). Data were statistically tested using ordinary one-way ANOVA by Prism 10. ^***^*P* < 0.001.
