# Supplementary figures and images for: Urolithin A and nicotinamide riboside differentially regulate innate immune defenses and metabolism in human microglial cells
Source: Front Aging Neurosci. 2024 Nov 27;16:1503336. doi: 10.3389/fnagi.2024.1503336 (PMC11631940; doi:10.3389/fnagi.2024.1503336)

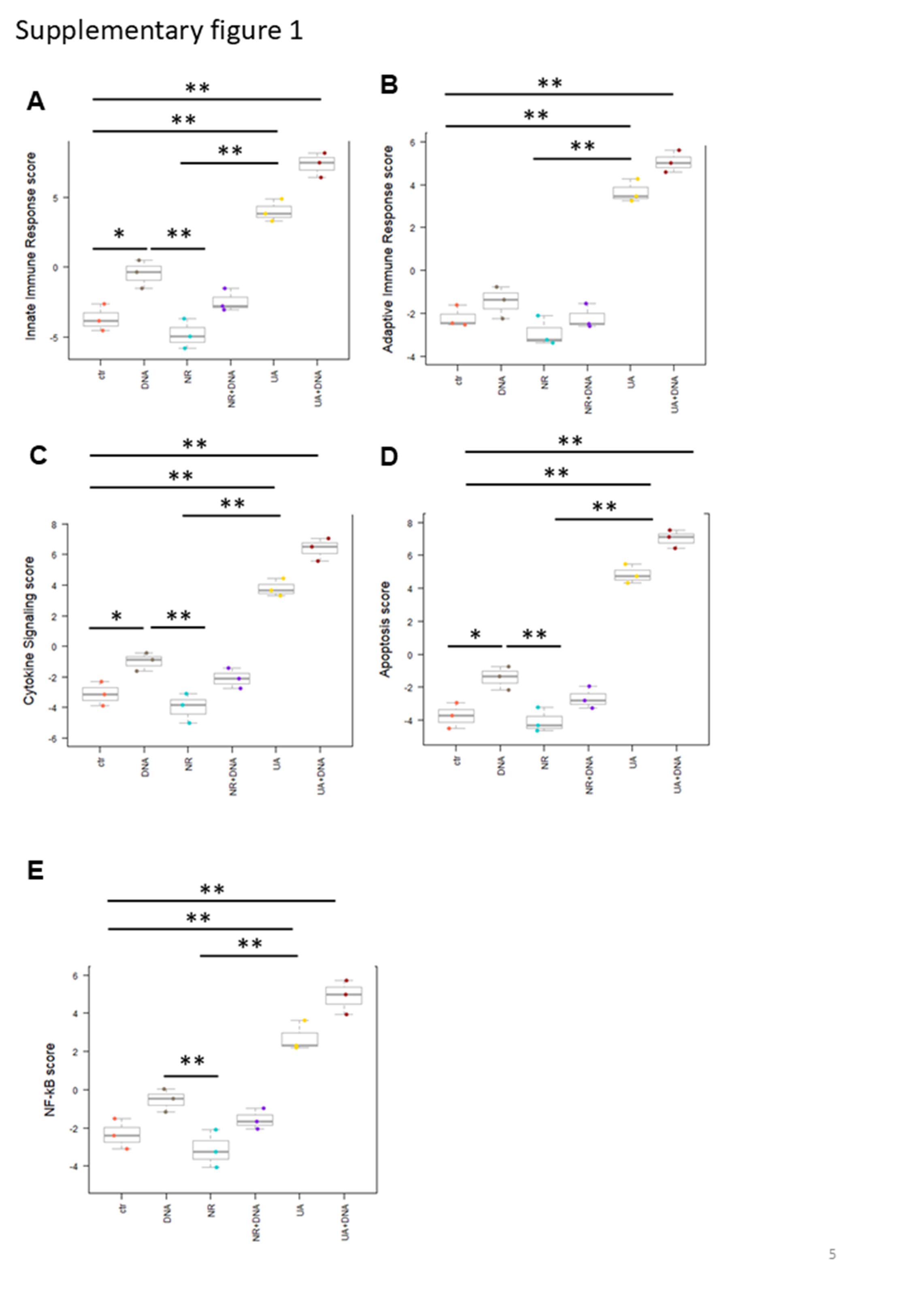

Supplement: Supplementary Figure S1 — NanoString pathway scores from the analysis in Figure 2A following UA, NR, and/or DNA stimulation of HMC3 cells for the pathways Innate Immune Response (A), Adaptive Immune Response (B), Cytokine Signalling (C), Apoptosis (D) and NF-kB (E). [file Image_1.tif]

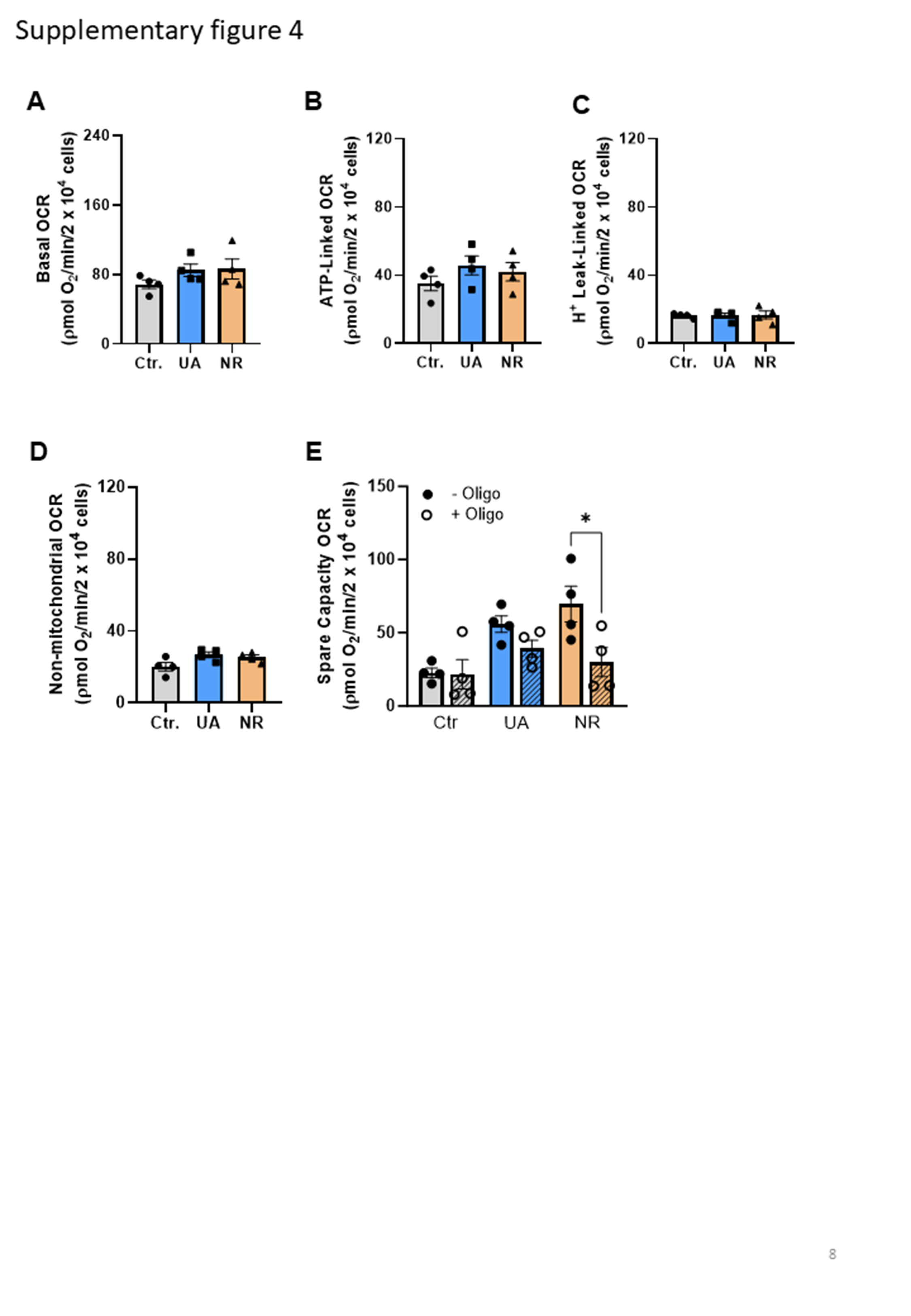

Supplement: Supplementary Figure S4 — Mitochondrial bioenergetics parameters and the effect of oligomycin in the Spare Capacity OCR in microglia cells treated with either UA or NR for 1 week. HMC3 cells (2.2 x 10^4^ cells/well), untreated (Ctr.) or treated with 10 μM UA or 3 mM NR for 1 week, were incubated in appropriate medium containing 2 mM glutamine and 11 mM glucose as metabolic energy substrates for OCR measurement. Different OCR parameters were determined: (A) Basal OCR, (B) ATP-Linked dependent OCR, (C) H+ Leak-linked OCR, and (D) Non-mitochondrial OCR. Panel E shows the effects of oligomycin on the underestimation of Spare Capacity OCR in cells subjected to different treatments. Each dot represents an independent experiment, and bars denote means (+ SEM) (N = 4). Data were statistically tested using ordinary one-way ANOVA by Prism 10. ***P < 0.001. [file Image_4.tif]
