# Supplementary material for: Urolithin A and nicotinamide riboside differentially regulate innate immune defenses and metabolism in human microglial cells
Source: Front Aging Neurosci. 2024 Nov 27;16:1503336. doi: 10.3389/fnagi.2024.1503336 (PMC11631940; doi:10.3389/fnagi.2024.1503336)
Supplement: Supplementary file 5 [file Supplementary_file_5.pdf]

Macro for β-galactosidase quantitation in ImageJ:

```
orgtitle=getTitle();
run("Duplicate...", " ");
title=getTitle();
run("Split Channels");
selectImage(title + " (red)");
run("Duplicate...", " ");
setThreshold(0, 77);
setOption("BlackBackground", false);
run("Convert to Mask");
run("Set Measurements...", "area integrated redirect=None decimal=5");
run("Analyze Particles...", "size=0-Infinity pixel show=[Overlay Masks] exclude add in_situ");
selectImage(title + " (red)");
run("From ROI Manager");
roiManager("measure");

for (k = 0; k < nResults; k + +)
{  area + = getResult('Area', k);
  Int + = getResult('IntDen', k);}

selectWindow(orgtitle);
run("HSB Stack");
run("Stack to Images");
selectWindow("Saturation");
run("Gaussian Blur...", "sigma=2");
run("Invert LUT");
setAutoThreshold("Otsu");

run("Convert to Mask");
run("Watershed");
roiManager("deselect");
roiManager("delete");
run("Analyze Particles...", "size=100-Infinity pixel show=[Overlay Masks] add in_situ");

selectWindow("Brightness");
run("From ROI Manager");
run("Clear Results");
roiManager("Measure");

for (k = 0; k < nResults; k + +)
{  MA + = getResult('Area', k);}
{ AMA = (MA/k);//calculates mean Area}

//deletes meassurements from Dapi ch which are unnecessary

nuc = roiManager("count");

MeanArea = area/nuc;
MeanInt = Int/nuc;
```
